# Urinary Calprotectin and Posttransplant Renal Allograft Injury

**DOI:** 10.1371/journal.pone.0113006

**Published:** 2014-11-17

**Authors:** Martin Tepel, Christoffer Borst, Claus Bistrup, Niels Marcussen, Nikolaos Pagonas, Felix S. Seibert, Robert Arndt, Walter Zidek, Timm H. Westhoff

**Affiliations:** 1 Department of Nephrology, Odense University Hospital, and University of Southern Denmark, Institute of Molecular Medicine, Cardiovascular and Renal Research, Institute of Clinical Research, Odense, Denmark; 2 Department of Pathology, Odense University Hospital, and University of Southern Denmark, Odense, Denmark; 3 Department of Nephrology, Charité, Campus Benjamin Franklin, Berlin, Germany; 4 Medizinische Klinik I, Univ.-Klinik Marienhospital Herne, Ruhr-University Bochum, Bochum, Germany; University of Toledo, United States of America

## Abstract

**Objective:**

Current methods do not predict the acute renal allograft injury immediately after kidney transplantation. We evaluated the diagnostic performance of urinary calprotectin for predicting immediate posttransplant allograft injury.

**Methods:**

In a multicenter, prospective-cohort study of 144 incipient renal transplant recipients, we postoperatively measured urinary calprotectin using an enzyme-linked immunosorbent assay and estimated glomerular filtration rate (eGFR) after 4 weeks, 6 months, and 12 months.

**Results:**

We observed a significant inverse association of urinary calprotectin concentrations and eGFR 4 weeks after transplantation (Spearman r = −0.33; P<0.001). Compared to the lowest quartile, patients in the highest quartile of urinary calprotectin had an increased risk for an eGFR less than 30 mL/min/1.73 m^2^ four weeks after transplantation (relative risk, 4.3; P<0.001; sensitivity, 0.92; 95% CI, 0.77 to 0.98; specificity, 0.48; 95% CI, 0.31 to 0.66). Higher urinary calprotectin concentrations predicted impaired kidney function 4 weeks after transplantation, as well as 6 months and 12 months after transplantation. When data were analyzed using the urinary calprotectin/creatinine-ratio similar results were obtained. Urinary calprotectin was superior to current use of absolute change of plasma creatinine to predict allograft function 12 months after transplantation. Urinary calprotectin predicted an increased risk both in transplants from living and deceased donors. Multivariate linear regression showed that higher urinary calprotectin concentrations and older donor age predicted lower eGFR four weeks, 6 months, and 12 months after transplantation.

**Conclusions:**

Urinary calprotectin is an early, noninvasive predictor of immediate renal allograft injury after kidney transplantation.

## Introduction

The immediate function of a renal allograft within the first weeks after kidney transplantation may vary considerably. The renal allograft may immediately start urine production. In contrast, the renal allograft may be damaged. An impaired renal allograft function within the first weeks or within the first year is detrimental to the longevity of the allograft and accounts for a 40% decrease in long-term graft survival [1]–[5]. A major role of ischemia-reperfusion injury for impaired renal allograft function has been confirmed in the literature [6].

Current methods do not predict the acute renal allograft injury immediately after kidney transplantation [7]. Recently, urinary calprotectin concentrations have been identified as a marker of acute kidney injury in the non-transplant population [8], [9]. Calprotectin is a calcium-binding complex of two proteins of the so-called S100 group. It serves as a mediator protein of the innate immune system. Calprotectin is derived predominantly from neutrophils and mononuclear cells and activates Toll-like receptor 4 thereby amplifying inflammatory activity [10]. The innate immune system plays a crucial role in the pathophysiology of ischemia-reperfusion injury in the kidney. Neutrophils and macrophages migrate into the transplant within 6 h of reperfusion and release proinflammatory cytokines and other soluble inflammatory mediators [11]. After ischemia-reperfusion injury of the tubular epithelial cells, Toll-like receptor 4 maintains and amplifies the inflammatory response [12]. In the present study we tested the hypothesis that urinary calprotectin at the first postoperative days predicts acute renal allograft injury and allograft function, i.e. estimated glomerular filtration rate (eGFR) 4 weeks, 6 months, and 12 months after transplantation.

## Materials and Methods

### Approval

The study protocol was in accordance with the ethical standards of the Declarations of Helsinki and Istanbul. The study was approved by the local ethics committees (Den Videnskabsetiske Komite for Region Syddanmark, Projekt-ID: 8-20100098 and Ethik-Komitee Charité Universitätsmedizin Berlin).

### Study population

We performed a multicenter prospective-cohort study of 144 patients receiving kidney transplants. Patients who were at least 18 years old and who were scheduled to receive living donor kidney transplants or deceased donor kidney transplants were recruited. Written informed consent was obtained from all patients before entry into the study. Baseline characteristics of donors and recipients and information on organ procurement were prospectively obtained from medical records. Induction therapy, immunosuppressive therapy, concomitant medications, and transplant biopsy were all made by the clinicians at each institution according to their local protocols. Delayed graft function was defined by at least one dialysis session within 7 days of transplantation [13]. Hemodialysis within 1 week after transplantation was performed because of uremic symptoms (29% of cases), hypervolemia causing dyspnoe (50% of cases), hyperkalemia (21% of cases). The treating physicians were unaware of the calprotectin concentrations. During follow up, one recipient died during the first year posttransplant due to a cardiovascular event. Six recipients lost graft function during the first year posttransplant. eGFR was available after 12 months in 123 patients.

### Enzyme-linked immunosorbent assay for urinary calprotectin and urinary kidney injury molecule-1

We collected one urine sample postoperatively. We aliquoted urine supernatants into cryovials, labeled each with a random barcode, and stored samples at −20°C. The enzyme-linked immunosorbent assay (ELISA) for urinary calprotectin was performed as previously published by our group using the PhiCal-Calprotectin assay kit (Immundiagnostik AG, Bensheim, Germany) according to the manufacturer’s protocol [8], [9]. Calprotectin concentrations were measured by personnel blinded to patient information. The coefficient of variation was less than 6% Urinary kidney injury molecule-1 (KIM-1) concentrations was measured using ELISA kits according to the manufacturer’s protocol (dianova, Hamburg, Germany). The coefficient of variation was less than 10%.

### Assessment of allograft function

Plasma creatinine was routinely measured. The absolute change in plasma creatinine was calculated as plasma creatinine preoperatively minus the first postoperative day. Four weeks, 6 months, and 12 months after transplantation we determined estimated glomerular filtration rate (eGFR) in kidney recipients according to the Chronic Kidney Disease Epidemiology Collaboration (CKD-EPI) equation [14].

eGFR = 141×min(Cr/κ,1)^α^×max(Cr/κ,1)^−1.209^×0.993^Age^×1.018 [if female]×1.159 [if black], where Cr is plasma creatinine in mg/dL, κ is 0.7 for females and 0.9 for males, α is −0.329 for females and −0.411 for males, min indicates the minimum of Cr/κ or 1, and max indicates the maximum of Cr/κ or 1.

CKD-EPI equation is recommended in transplanted patients because it more accurately gives true glomerular filtration rate measured by plasma clearance of (99 m)Tc-diethylenetriamine pentaacetic acid [15].

### Histological findings

In 98 patients a baseline biopsy was performed at transplantation as benchmark for comparison of post-transplantation histology in case a post-transplant indication biopsy was necessary. Median number of glomeruli in the baseline biopsy was 7 (IQR, 3 to 11). The severity of lesions (arteriolar hyalinosis, arteriolosclerosis, interstitial fibrosis) was scored semiquantitatively as published [16], [17].

### Statistics

Continuous data are presented as median and interquartile range (IQR). Non-parametric Mann-Whitney test or Kruskal-Wallis test with Dunn’s Multiple Comparison Test were used as appropriate to detect differences between the groups. Frequency counts were calculated for categorical data. Differences in these categorical variables between the groups were analyzed by Chi-square test or Fisher’s exact text as appropriate. Associations between variables were determined using non-parametric Spearman correlation. We performed receiver operating characteristic (ROC) curve analysis to detect the accuracy of urinary calprotectin, urinary KIM-1, and absolute change in plasma creatinine for predicting eGFR after transplantation.

We performed linear regression for associations between urinary calprotectin and eGFR four weeks after kidney transplantation while adjusting for donor age, donor gender, living-donor vs. deceased-donor status, recipient age, recipient gender, delayed graft function, recipient months on dialysis before transplantation, and use of prednisolone. For regression analyses urinary calprotectin concentrations were log-transformed to obtain normal distributed data to obtain normal distributed data as tested by Kolmogoroff-Smirnov-test. Multivariate models were constructed with backward variable selection, using P<0.05 for variable retention.

Data were analyzed using GraphPad prism software (version 5.0, GraphPad Software, San Diego, CA, USA) and SPSS for windows (version 15.0; SPSS, Chicago, IL, USA). All statistical tests were two-sided. Two-sided p-values less than 0.05 were considered to indicate statistical significance.

## Results

### Characteristics of cohort at baseline

Urinary calprotectin concentrations were measured in 144 patients after renal allograft transplantation. 85 transplant recipients were male (59%), and 59 were female (41%). Median age of recipients was 52 years (IQR, 45 to 59 years). The cause of chronic kidney disease was diabetic nephropathy in 14 cases (10%), hypertensive nephropathy in 19 cases (13%), chronic glomerulonephritis in 53 cases (37%), polycystic kidney disease in 22 cases (15%), and other/unknown in 36 cases (25%). The number of patients with second or more transplants was 17 (12%). Median time on dialysis before transplantation was 26 months (IQR, 4 to 71 months). 31 patients (22%) were smokers, 125 patients (87%) had hypertension, and 15 patients (10%) had a history of cardiovascular events. There was no association of calprotectin and cardiac events (Spearman r = 0.08; 95% CI, 0.08 to 0.25; P = 0.31). 68 patients (47%) received kidneys from living donors, and 76 patients (53%) from deceased donors. 101 patients (70%) received tacrolimus, 43 patients (30%) cyclosporine A, 144 patients (100%) mycophenolate acid, and 75 patients (52%) prednisolone, respectively. The clinical characteristics of patients and their allograft are shown in **Table 1**.

**Table 1:** Clinical characteristics of 144 patients with renal allograft.

Characteristic	All patients	Q1	Q2	Q3	Q4	P-value
Age of the donor	52	51	56	51	52	0.13
(years)	(45–59)	(45–57)	(49–66)	(46–57)	(43–67)	
Deceased kidney	76	11	19	16	30	<0.001
(number (%))	(53%)	(31%)	(53%)	(44%)	(83%)	
Cold-ischemia time	600	398	754	415	607	0.26
(min)	(290–840)	(163–840)	(293–914)	(166–664)	(447–798)	
HLA mismatches	3	3	3	3	3	0.37
(range, 0 to 6)	(2–4)	(2–4)	(3–4)	(2–4)	(2–4)	
Age of recipient	52	52	53	50	53	0.18
(years)	(45–59)	(40–61)	(39–61)	(42–58)	(48–65)	
Gender male, n (%)	85 (59)	22 (61)	22 (61)	19 (53)	22 (61)	0.86
Duration of dialysis	26	5	29	22	72	<0.001
(months)	(4–71)	(0–32)	(5–68)	(7–59)	(25–90)	
Body weight (kg)	79 (66–89)	74 (64–89)	82 (69–91)	79 (68–97)	77 (65–89)	0.36
Body mass-index	26	24	25	26	26	0.10
(kg/m^2^)	(23–29)	(23–27)	(24–28)	(24–30)	(23–29)	
Systolic blood	148	150	152	145	144	0.19
pressure (mmHg)	(130–161)	(135–166)	(136–170)	(124–154)	(128–169)	
Diastolic blood	84	85	87	80	80	0.75
pressure (mmHg)	(76–94)	(76–97)	(78–92)	(75–97)	(71–94)	

Continuous data are presented as median (interquartile range). Data are shown for all patients and according to quartiles (Q) of urinary calprotectin concentrations (with Q1, less than 286 ng/mL; Q2, 286 to 576 ng/mL; Q3, 577 to 1694 ng/mL; Q4, more than 1694 ng/mL). Categorical data are presented as numbers (percent). Groups containing continuous data were compared using Kruskal-Wallis test, whereas groups containing categorical data were compared using Chi-squared test.

### Urinary calprotectin concentrations predict allograft function

**Figure 1:**
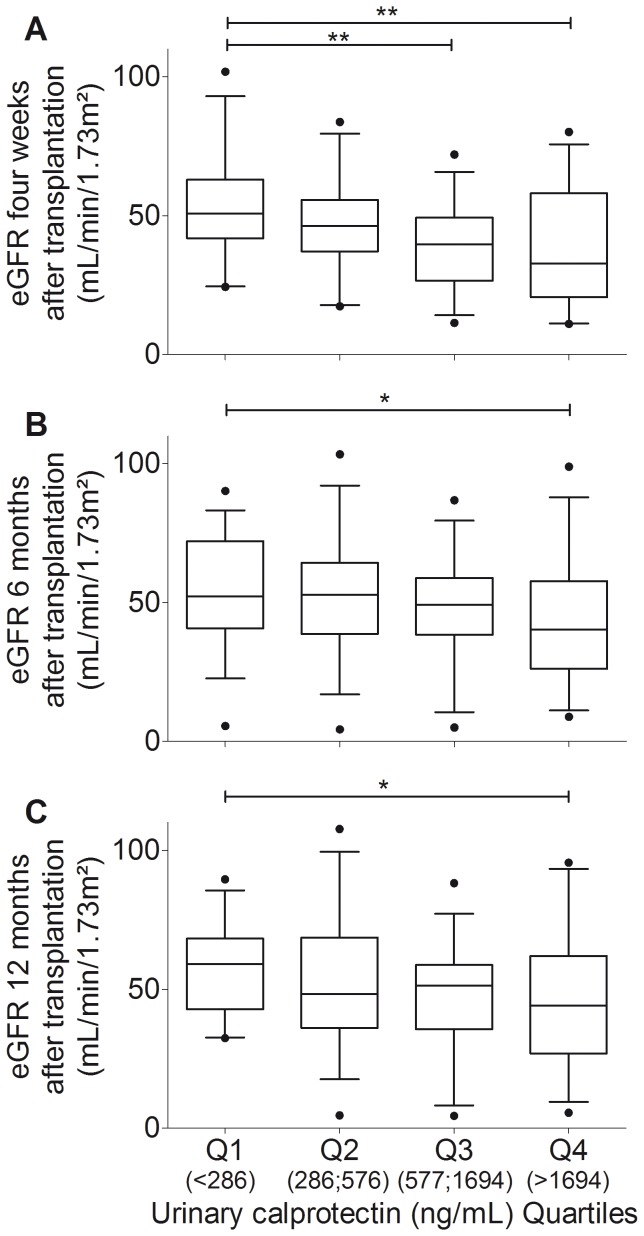
Urinary calprotectin concentrations predict renal allograft function. Box-and-whiskers-plots showing the reduction of estimated glomerular filtration rate (eGFR) four weeks (**A**), 6 months (**B;** n = 135), and 12 months (**C;** n = 123) after transplantation with increasing quartiles (Q) of urinary calprotectin concentrations. Boxes indicate median and interquartile ranges, whiskers indicate 5 to 95 percentile. Significant differences between the shown groups were **P<0.01 and *P<0.05 by Kruskal-Wallis test and Dunn’s multiple comparison test.

The median urinary calprotectin concentration was 576 ng/mL (IQR, 286 to 1694 ng/mL). In the lowest calprotectin quartile eGFR was 51 mL/min/1.73 m^2^ (IQR, 42 to 63 mL/min/1.73 m^2^), then eGFRs were 46 mL/min/1.73 m^2^ (IQR, 37 to 56 mL/min/1.73 m^2^) and 40 mL/min/1.73 m^2^ (IQR, 30 to 49 mL/min/1.73 m^2^), and finally in the highest calprotectin quartile eGFR was 33 mL/min/1.73 m^2^ (IQR, 21 to 58 mL/min/1.73 m^2^) (P = 0.004; **Figure 1A**). 6 months after transplantation eGFRs were 52 mL/min/1.73 m^2^ (IQR, 41 to 72 mL/min/1.73 m^2^) and 40 mL/min/1.73 m^2^ (IQR, 26 to 58 mL/min/1.73 m^2^) in the lowest and highest calprotectin quartile, respectively (P<0.05; **Figure 1B**). 12 months after transplantation eGFRs were 59 mL/min/1.73 m^2^ (IQR, 43 to 68 mL/min/1.73 m^2^) and 44 mL/min/1.73 m^2^ (IQR, 27 to 62 mL/min/1.73 m^2^) in the lowest and highest calprotectin quartile, respectively (P<0.05; **Figure 1C**).

**Figure 2:**
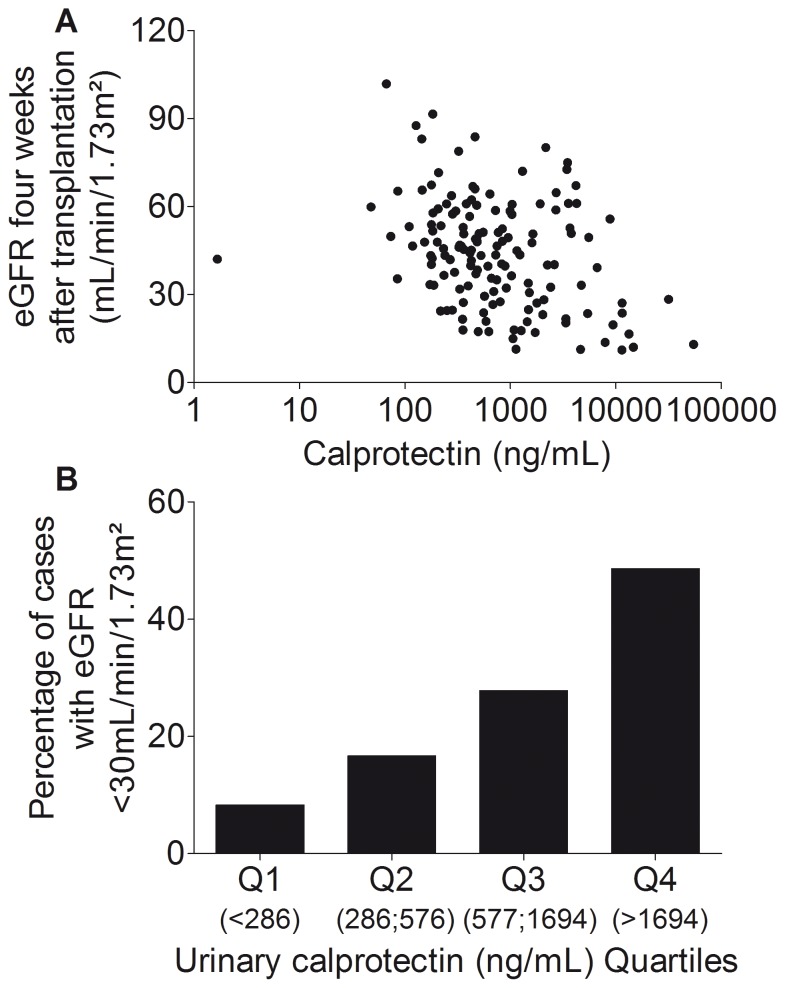
Association of urinary calprotectin with estimated glomerular filtration rate. **A.** Dot plot depicting the significant association of urinary calprotectin levels (log scale) with estimated glomerular filtration rate (eGFR) four weeks after transplantation. Spearman r = −0.33; P<0.001. **B.** Percentage of cases with estimated glomerular filtration rate (eGFR) after 4 weeks in the lowest quartile (i.e., less than 30 mL/min/1.73 m^2^) is shown. Bar graph shows percentages of cases from the contingency table, therefore there are no error bars. Chi-square derived from the contingency table was 17.10; P<0.001.

We observed a significant association of urinary calprotectin levels with eGFR 4 weeks after transplantation (Spearman r = −0.33; 95% CI, −0.47 to −0.17; P<0.001; **Figure 2A**), 6 months after transplantation (Spearman r = −0.20; 95% CI, −0.36 to −0.03; P = 0.018), and 12 months after transplantation (Spearman r = −0.22; 95% CI, −0.39 to −0.04; P = 0.015).

**Figure 2B** indicates that 48.6% of patients with urinary calprotectin concentrations in the highest quartile (i.e., more than 1694 ng/mL) showed an eGFR in the lowest quartile (i.e., less than 30 mL/min/1.73 m^2^) 4 weeks after renal transplantation. On the other hand, only 8.3% of patients with urinary calprotectin concentrations in the lowest quartile (i.e., less than 286 ng/mL) showed an eGFR in the lowest quartile (relative risk, 4.3; P<0.001; sensitivity, 0.92; 95% CI, 0.77 to 0.98; specificity, 0.48; 95% CI, 0.31 to 0.66). As the likelihood ratio equals 1.78, patients with higher urinary calprotectin concentrations more likely showed worse renal function.

The predictive effect of urinary calprotectin could be observed both in transplants from living donors and deceased donors. In transplants from living donors the relative risk was 4.9 (P = 0.039; sensitivity, 0.94; 95% CI, 0.71 to 1.00; specificity, 0.41; 95% CI, 0.18 to 0.67; likelihood ratio, 1.60). In transplants from deceased donors the relative risk was 3.5 (P = 0.030; sensitivity, 0.89; 95% CI, 0.67 to 0.99; specificity, 0.47; 95% CI, 0.24 to 0.71; likelihood ratio, 1.70).

Receiver-operating characteristic (ROC) curves showed that urinary calprotectin was able to predict the eGFR in the lowest quartile (i.e., less than 30 mL/min/1.73 m^2^) 4 weeks after renal transplantation. Urinary calprotectin resulted in an area under curve (AUC) of 0.75 (95% CI, 0.66 to 0.84; P<0.001).

**Figure 3:**
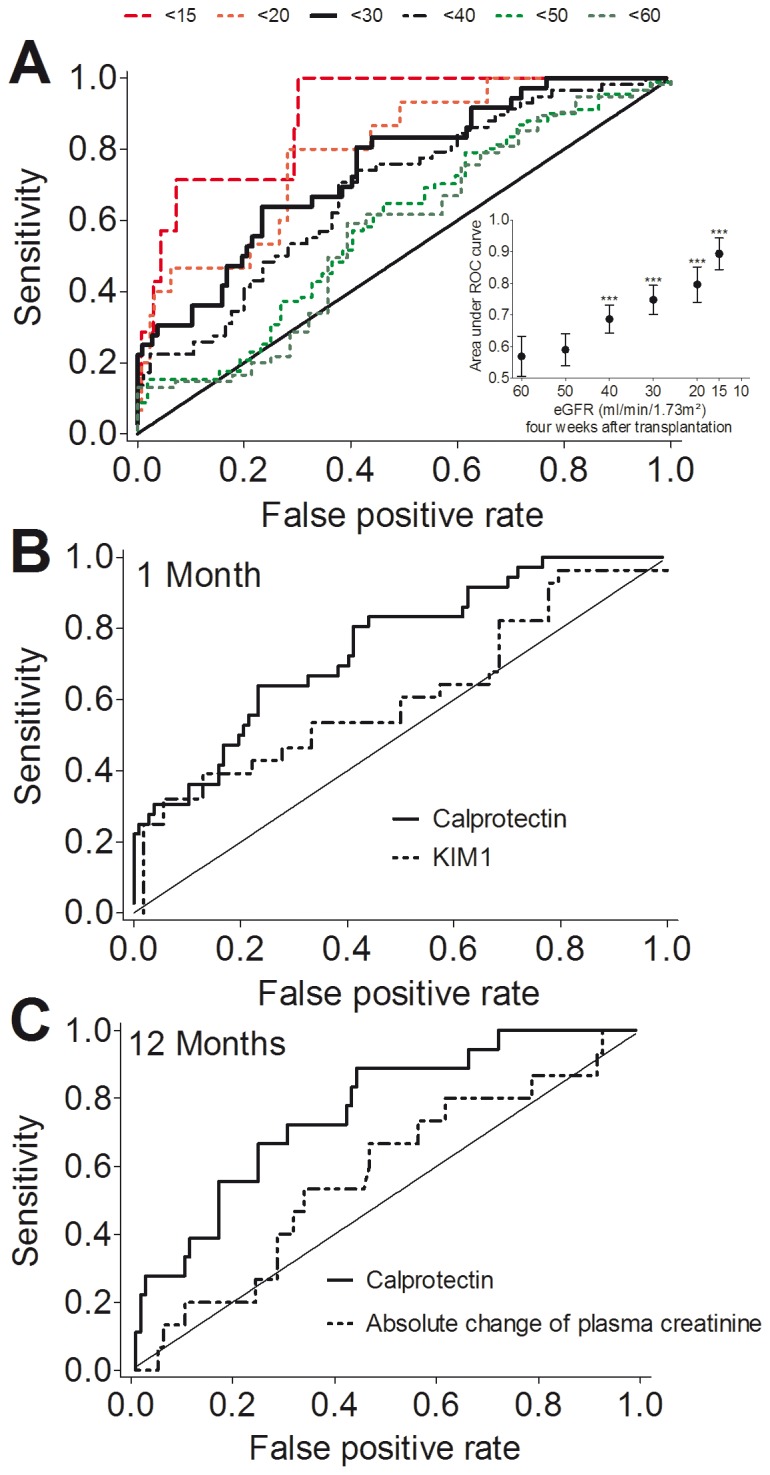
Calprotectin predicts kidney function. **A.** Receiver-operating characteristic (ROC) curves and summary data (**Inset**) for area under curve showing that urinary calprotectin concentrations predicted impaired kidney function 4 weeks after transplantation. All analyses were performed in the entire population. eGFR after 4 weeks in mL/min/1.73 m^2^ are indicated in the legend above the curves. The inset indicates each area under curve per eGFR curve. ***P<0.001. **B.** Receiver-operating characteristic (ROC) curves for urinary calprotectin and urinary kidney injury molecule-1 (KIM-1) concentrations for predicting the eGFR in the lowest quartile (i.e., less than 30 mL/min/1.73 m^2^) 1 month after renal transplantation. **C.** Receiver-operating characteristic (ROC) curves for urinary calprotectin and absolute change of plasma creatinine for predicting the eGFR in the lowest quartile (i.e., less than 30 mL/min/1.73 m^2^) 12 months after renal transplantation.

**Figure 3A** shows ROC curves and summary data for the AUC according to eGFR 4 weeks after transplantation. Higher values for area under curve were observed with lower eGFR 4 weeks after transplantation, ranging from 0.89 (95% CI, 0.80 to 0.99; P<0.001) with eGFR less than 15 mL/min/1.73 m^2^ (n = 7) to 0.69 (95% CI, 0.60 to 0.77; P<0.001) with eGFR less than 40 mL/min/1.73 m^2^ (n = 58).

When data were analyzed using the urinary calprotectin/creatinine-ratio similar results were obtained: The median urinary calprotectin/creatinine-ratio was 1.15 ng/g (IQR 0.67 to 3.00 ng/g). In the lowest calprotectin/creatinine quartile eGFR 4 weeks after renal transplantation was 46 mL/min/1.73 m^2^ (IQR, 34 to 64 mL/min/1.73 m^2^) whereas the highest quartile showed an eGFR of 36 mL/min/1.73 m^2^ (IQR, 21 to 55 mL/min/1.73 m^2^; P = 0.041). Receiver-operating characteristic (ROC) curves showed that urinary calprotectin/creatinine ratio was able to predict the eGFR in the lowest quartile (i.e., less than 30 mL/min/1.73 m2) 4 weeks after renal transplantation (AUC, 0.66; 95% CI, 0.55 to 0.77; P = 0.004).

For comparison, data were obtained using urinary kidney injury molecule-1 (KIM-1). The median urinary KIM-1 was 0.56 ng/mL (IQR, 0.29 to 1.14 ng/mL). In the lowest KIM-1 quartile eGFR was 48 mL/min/1.73 m^2^ (IQR, 28 to 60 mL/min/1.73 m^2^), then eGFRs were 34 mL/min/1.73 m^2^ (IQR, 24 to 45 mL/min/1.73 m^2^) and 40 mL/min/1.73 m^2^ (IQR, 33 to 51 mL/min/1.73 m^2^), and finally in the highest KIM-1 quartile eGFR was 28 mL/min/1.73 m^2^ (IQR, 18 to 44 mL/min/1.73 m^2^) (P = 0.06). 6 months after transplantation eGFRs were 54 mL/min/1.73 m^2^ (IQR, 38 to 62 mL/min/1.73 m^2^) and 34 mL/min/1.73 m^2^ (IQR, 25 to 58 mL/min/1.73 m^2^) in the lowest and highest KIM-1 quartile, respectively (P = 0.17). 12 months after transplantation eGFRs were 51 mL/min/1.73 m^2^ (IQR, 40 to 60 mL/min/1.73 m^2^) and 32 mL/min/1.73 m^2^ (IQR, 27 to 57 mL/min/1.73 m^2^) in the lowest and highest KIM-1 quartile, respectively (P = 0.25). Urinary KIM-1 levels were associated with eGFR 4 weeks after transplantation (Spearman r = −0.23; P = 0.034). We observed that 55.0% of patients with urinary KIM-1 in the highest quartile (i.e., more than 1.14 ng/mL) showed an eGFR in the lowest quartile (i.e., less than 30 mL/min/1.73 m^2^) 4 weeks after renal transplantation, and 25.0% of patients with urinary KIM-1 in the lowest quartile (i.e., less than 0.29 ng/mL) showed an eGFR in the lowest quartile (relative risk, 2.0; P = 0.11). As depicted in **Figure 3B** KIM-1 resulted in an AUC of 0.61 (95% CI, 0.48 to 0.75; P = 0.09), indicating that calprotectin, but not KIM-1 predicted the eGFR in the lowest quartile (i.e., less than 30 mL/min/1.73 m^2^).

We observed that urinary calprotectin was superior to current use of absolute change of plasma creatinine to predict allograft function. Urinary calprotectin resulted in an AUC of 0.76 (95% CI, 0.65 to 0.88; P<0.001) to predict eGFR less than 30 mL/min/1.73 m^2^ 12 months after transplantation, whereas the absolute change of plasma creatinine resulted in an AUC of 0.57 (95% CI, 0.42 to 0.72; P = 0.38) (**Figure 3C**).

Univariate and multivariate linear regression analyses are shown in **Tables 2** **and** **3**. Multivariate linear regression showed that higher urinary calprotectin concentrations (P<0.001), older donor age (P<0.001), and delayed graft function (P = 0.039) predicted lower eGFR four weeks after transplantation, but not donor gender, living-donor vs. deceased-donor status, recipient age, recipient gender, recipient months on dialysis before transplantation, or prednisolone. As shown in **Table S1** using mixed model analyses for estimated glomerular filtration rate 4 weeks after kidney transplantation confirmed these results. We also found that that higher urinary calprotectin concentrations and older donor age predicted lower eGFR 6 months as well as 12 months after transplantation.

**Table 2:** Univariate linear regression analyses for estimated glomerular filtration rate 4 weeks after kidney transplantation.

Variable	B	SE of B	P	95% CI of B
Urinary calprotectin	−8.391	2.432	0.001	−13.201 to −3.580
Donor age	−0.577	0.120	<0.001	−0.814 to −0.339
Donor gender (0 = female; 1 = male)	5.168	2.755	0.063	−0.283 to 10.619
Donor status (0 = LD; 1 = DD)	−3.308	3.493	0.345	−10.219 to 3.603
Recipient age	−0.040	0.117	0.735	−0.271 to 0.192
Recipient gender (0 = female; 1 = male)	1.212	2.801	0.666	−4.329 to 6.753
Delayed graft function (0 = no DGF; 1 = DGF)	−5.638	3.409	0.101	−12.382 to 1.106
Duration of dialysis before transplantation (months)	−0.005	0.040	0.897	−0.084 to 0.074
Prednisolone (0 = no; 1 = yes)	2.002	3.223	0.536	−4.374 to 8.379

B indicates regression coefficient; SE indicates standard error; CI indicates confidence interval. Calprotectin was analyzed as a continuous variable. Regression analyses were performed after logarithmic transformation of urinary calprotectin concentrations.

LD indicates living donor; DD indicates deceased donor. DGF indicates delayed graft function.

**Table 3:** Multivariate linear regression analyses for estimated glomerular filtration rate 4 weeks, 6 months and 12 months after kidney transplantation.

Variable	B	SE of B	P	95% CI of B
4 weeks after transplantation				
Urinary calprotectin	−9.636	2.199	<0.001	−13.985 to −5.287
Donor age	−0.571	0.115	<0.001	−0.797 to −0.344
Delayed graft function	−5.925	2.839	0.039	−11.539 to −0.312
6 months after transplantation				
Urinary calprotectin	−5.516	2.416	0.024	−10.295 to −0.737
Donor age	−0.808	0.127	<0.001	−1.058 to −0.557
12 months after transplantation				
Urinary calprotectin	−5.895	2.816	0.038	−11.470 to −0.318
Donor age	−0.735	0.148	<0.001	−1.027 to −0.443

B indicates regression coefficient; SE indicates standard error; CI indicates confidence interval. Calprotectin was analyzed as a continuous variable. Regression analyses were performed after logarithmic transformation of urinary calprotectin concentrations.

LD indicates living donor; DD indicates deceased donor. DGF indicates delayed graft function.

Median eGFR 4 weeks after transplantation were not significantly different between the subgroups without or with prednisolone (46 mL/min/1.73 m^2^; IQR, 33 to 53 mL/min/1.73 m^2^; vs. 43 mL/min/1.73 m^2^; IQR, 25 to 59 mL/min/1.73 m^2^; P = 0.66). Furthermore, median urinary calprotectin concentrations were not significantly different between the subgroups without or with prednisolone (552 ng/mL; IQR, 262 to 1260 ng/mL; vs. 638 ng/mL; IQR 302 to 2239 ng/mL; P = 0.29).

Patients with delayed graft function showed higher urinary calprotectin levels, however that difference was only marginally significant (819 ng/mL; IQR 346 to 2627 ng/mL; vs. 522 ng/mL; IQR, 262 to 1308 ng/mL; P = 0.09). Multivariate logistic regression showed that living-donor vs. deceased-donor status (P = 0.003) predicted delayed graft function. In contrast urinary calprotectin (P = 0.51), donor age (P = 0.54), donor gender (P = 0.68), recipient age (P = 0.57) as well as recipient gender (P = 0.19) did not show significant effects. For delayed graft function urinary calprotectin resulted in an AUC of 0.59 (95% CI, 0.49 to 0.69; P = 0.09). For delayed graft function urinary KIM-1 resulted in an AUC of 0.52 (95% CI, 0.39 to 0.65; P = 0.74).

We also analyzed urinary calprotectin levels in a subgroup of 38 patients who were anuric before renal transplantation. We would like to emphasize that that subgroup of 38 patients were anuric before renal transplantation, but not after transplantation. 5 out of 10 previously anuric patients (50.0%) with urinary calprotectin concentrations in the highest quartile showed a creatinine clearance in the lowest quartile. On the other hand, none out of 10 previously anuric patients (0.0%) with urinary calprotectin concentrations in the lowest quartile showed a creatinine clearance in the lowest quartile (P = 0.03).

### Histological findings

Histological evaluation could be performed in 98 baseline biopsies obtained at the time of transplantation. Urinary calprotectin concentrations were similar in patients receiving a graft without or with arteriolar hyalinosis (grade 0, median, 426 ng/mL (IQR, 233 to 1643 ng/mL); vs. grade >0, median, 659 ng/mL (IQR, 357 to 1260 ng/mL; P = 0.29)). Urinary calprotectin concentrations were also similar in patients receiving a graft without or with arteriolosclerosis (grade 0, median, 463 ng/mL (IQR, 238 to 1204 ng/mL) vs. grade 1, median, 616 ng/mL (IQR, 355 to 1191 ng/mL; P = 0.52)) and receiving a graft without or with interstitial fibrosis (grade 0, median, 478 ng/mL (IQR, 241 to 1476 ng/mL) vs. grade 1, median, 724 ng/mL (IQR, 390 to 1024 ng/mL; P = 0.59)). Only 13 patients had suspected rejection during the first 4 postoperative weeks, only 1 patient showed BANFF grade 1A rejection. These data may indicate that preexisting renal lesions per se may not affect postoperative urinary calprotectin concentrations.

## Discussion

In the present study we showed that postoperative urinary calprotectin predicts kidney allograft function after transplantation. We observed that use of urinary calprotectin was superior to current use of absolute change of plasma creatinine to predict allograft function after transplantation. It is noteworthy to acknowledge the biologically plausible role of calprotectin in the mechanisms of ischemic reperfusion injury after kidney transplantation. The tubular damage leads to a secondary activation of the innate immune system with immigration of granulocytes and monocytes. Moreover it leads to an amplification and activation of toll-like receptors [18]. Calprotectin is a ligand of toll-like receptors, which is constitutively expressed in both proximal and distal tubules [19]. Hall et al. [20] reported that neutrophil gelatinase-associated lipocalin predicted dialysis within one week after kidney transplantation. They reported that 34 out of 91 patients (37%) had delayed graft function, defined by at least one dialysis session within the first postoperative week [20]. However, it is well-known that the clinical decision to perform postoperative hemodialysis may be influenced by several factors beyond renal allograft function, for example fluid overload and clinical uremic status of the patient.

Looking at the results for urinary calprotectin and urinary KIM-1 it may be speculated that an increased neutrophil and macrophage response to ischemia-reperfusion injury may cause increased calprotectin levels. This may be supported by the finding that urinary calprotectin but not urinary KIM-1 was significantly associated with renal allograft function.

A study by Szeto et al. in 63 kidney transplant recipients investigated urinary mRNA of neutrophil gelatinase-associated lipocalin, kidney injury molecule-1, interleukin-18, surfactant protein-C, and S100 calcium-binding proteins A8 and A9 according to histologic groups observed in biopsies from biopsy because of progressive worsening of allograft function. They showed that urinary mRNA of KIM-1 may provide prognostic information about renal function decline, irrespective of the kidney pathology [21]. No data are available for urinary calprotectin protein and pathology because of progressive worsening of allograft function.

The present study confirms previous reports that donor age has a large impact on allograft function. However, there is no treatment to adjust for the fact of using allografts from older subjects. It should be noted that urinary calprotectin predicted allograft function even after adjustment for donor age. Hence, measurements of urinary calprotectin may add to the diagnostic procedures.

Beside the use of urinary calprotectin for prediction of immediate renal allograft injury, its determination immediately after kidney transplantation may be helpful and a necessary step for the implementation of future strategies to improve outcome after kidney transplantation. In summary, urinary calprotectin is an early, noninvasive predictor of acute renal allograft injury after kidney transplantation.

## Supporting Information

Table S1 **Mixed model analyses for estimated glomerular filtration rate 4 weeks after kidney transplantation.**
(DOC)
