# Supplementary material for: Urinary Calprotectin and Posttransplant Renal Allograft Injury
Source: PLoS One. 2014 Nov 17;9(11):e113006. doi: 10.1371/journal.pone.0113006 (PMC4234472; doi:10.1371/journal.pone.0113006)
Supplement: Table S1 — Mixed model analyses for estimated glomerular filtration rate 4 weeks after kidney transplantation. (DOC) [file pone.0113006.s001.doc]

**Supplemental material**

**Supplemental Table S1**. Mixed model analyses for estimated glomerular filtration rate 4 weeks after kidney transplantation.

| Variable | B | SE of B | P | 95% CI of B |
| --- | --- | --- | --- | --- |
| Urinary calprotectin | -7.777 | 2.505 | 0.002 | -12.732 to -2.822 |
| Donor age | -0.575 | 0.119 | <0.001 | -0.811 to -0.338 |
| Donor gender (0=female; 1=male) | 5.093 | 2.752 | 0.066 | -0.350 to 10.537 |
| Donor status (0=LD; 1=DD) | -2.917 | 3.444 | 0.399 | -9.731 to 3.897 |
| Recipient age | -0.064 | 0.118 | 0.589 | -0.297 to 0.169 |
| Recipient gender (0=female; 1=male) | 1.535 | 2.802 | 0.585 | -4.008 to 7.078 |
| Delayed graft function (0=no DGF; 1=DGF) | -6.132 | 3.325 | 0.067 | -12.711 to 0.447 |
| Duration of dialysis before transplantation (months) | -0.020 | 0.040 | 0.617 | -0.098 to 0.058 |
| Prednisolone (0=no; 1=yes) | 2.298 | 3.157 | 0.468 | -3.948 to 8.544 |

B indicates coefficient; SE indicates standard error; CI indicates confidence interval. Calprotectin was analyzed as a continuous variable. Analyses were performed after logarithmic transformation of urinary calprotectin concentrations.

LD indicates living donor; DD indicates deceased donor. DGF indicates delayed graft function.
